# Tuberculosis with Evans syndrome: A case report

**DOI:** 10.1002/ccr3.4113

**Published:** 2021-05-06

**Authors:** Sagar Gyawali, Utsav Joshi, Zeni Kharel, Shambhu Khanal, Anjan Shrestha

**Affiliations:** ^1^ Department of Internal Medicine Institute of Medicine Tribhuvan University Teaching Hospital Kathmandu Nepal; ^2^ Department of Internal Medicine Rochester General Hospital Rochester NY USA

**Keywords:** autoimmune hemolytic anemia, Evans syndrome, tuberculosis

## Abstract

Evans syndrome and tuberculosis could be predisposing factors for one another, or there may be a common pathophysiological denominator for the co‐occurrence. Further research is needed for a better understanding of pathophysiology and treatment.

## Graphical abstract

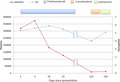

## 1 INTRODUCTION

We herein report an exceedingly rare case of Evans syndrome with associated tubercular pleural effusion. The patient was initially treated as autoimmune hemolytic anemia. However, the development of thrombocytopenia led to the subsequent diagnosis of Evans syndrome. The co‐existence of tuberculosis resulted in additional difficulty during treatment with immunosuppressive medications.

Evans syndrome (ES), a hematological entity described by Evans and colleagues in 1951, is characterized by the presence of Coombs positive hemolytic anemia and immune thrombocytopenia, and less commonly, autoimmune neutropenia.^1^ Although the exact pathophysiology of ES remains unknown, immune dysfunction with subsequent production of antibodies targeting the erythrocytes and platelets is a likely mechanism. Glucocorticoids and intravenous immunoglobulin (IVIG) have mostly been used as first‐line therapy for ES. Other treatment options include immunosuppressants, blood transfusion, splenectomy, and hematopoietic stem cell transplant.^2^ ES has a more variable clinical course as compared to isolated autoimmune hemolytic anemia (AIHA), with more frequent exacerbations and mortality.^3^ The coexistence of tuberculosis (TB) with Evans syndrome makes therapy even more challenging.

## 2 CASE PRESENTATION

A 20‐year‐old nonalcoholic, nonsmoker Nepalese man with no significant past medical history presented to the outpatient department with melena for 10 days. This was associated with fatigue and generalized weakness of the body. He denied any abdominal pain, nausea, vomiting, or recent weight loss. He also gave a history of low‐grade fever with an evening rise of temperature for the same duration. It was not associated with chest pain, cough, hemoptysis, runny nose, watery eyes, or sore throat. He had no history of similar illness in the past or any recent sick contacts. His family history was unremarkable.

Vitals on presentation revealed blood pressure 100/70 mm Hg, pulse 120/min, respiratory rate 24/min, and temperature 100.5°F. On physical examination, he was pale and icteric. There were no edema, ecchymosis, or any palpable lymph nodes. Chest auscultation revealed diminished breath sounds over the right infrascapular region. The rest of the systemic examination was normal.

Blood investigations showed hemoglobin (Hb) 5.3 g/dL and white blood cells (WBC) 5400/mm^3^ with neutrophils 79% and lymphocytes 18%, and platelets 319 000/mm^3^. His renal function tests showed urea 4.5 mmol/L and creatinine 95 μmol/L. Liver function test (LFT) showed total bilirubin 66 μmol/L, direct bilirubin 7 μmol/L, alanine aminotransferase (ALT) 23 U/L, aspartate aminotransferase (AST) 38 U/L, and alkaline phosphatase (ALP) 161 U/L. His lactate dehydrogenase (LDH) was elevated to 1450 U/L. Direct Coombs test was positive for antibodies against red blood cells.

Serologies were negative for human immunodeficiency virus (HIV), hepatitis B virus, and hepatitis C virus. Serological evaluation for antinuclear antibody (ANA), antidouble‐stranded deoxyribonucleic acid antibody (ds‐DNA), and anti–smooth muscle antibody (ASMA) were negative. Immunoglobulin levels were normal. A presumptive diagnosis of autoimmune hemolytic anemia was made, and the patient was started on oral prednisone 60 mg daily.

Due to the presenting complaint of melena, upper gastrointestinal endoscopy was done, which revealed a peptic ulcer in the first part of the duodenum. A tissue sample was taken during endoscopy, which did not reveal Helicobacter pylori. He was treated with proton pump inhibitors for his peptic ulcer disease.

**FIGURE 1:**
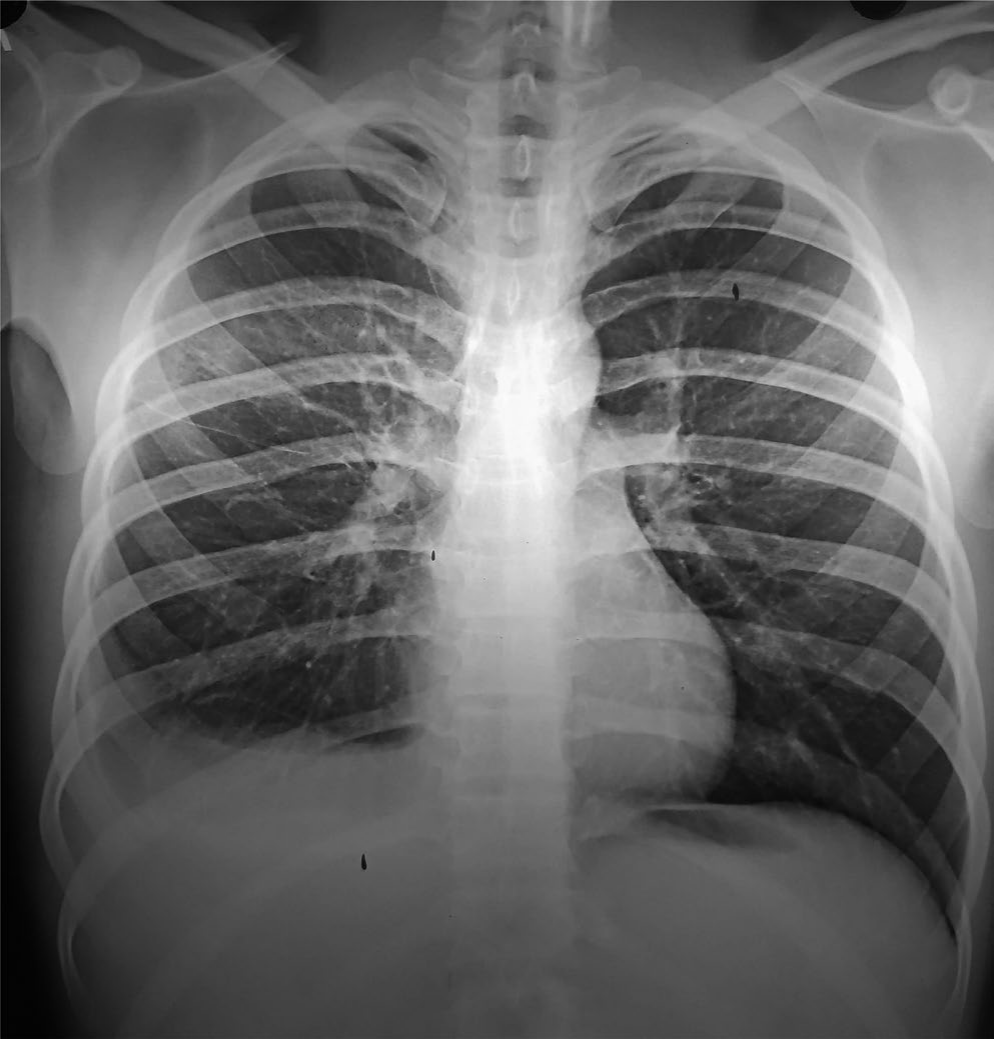
Chest x‐ray showing pleural effusion

Meanwhile, a chest X‐ray showed right‐sided pleural effusion (Figure 1). The pleural fluid analysis showed an exudative pleural effusion with lymphocyte predominance, lymphocyte to neutrophil ratio of 4, and high adenosine deaminase activity (89 units/L). Computed tomography scan (CT) of the chest with and without contrast showed multiple centriacinar nodules giving tree in bud appearance, fibrotic changes in the right upper lobe, and moderate right‐sided pleural effusion suggestive of tubercular pathology. Acid‐fast bacilli (AFB) was not visualized in the microscopic analysis of the sputum. However, due to high clinical suspicion, corroborative pleural fluid analysis and chest imaging findings, he was started on antitubercular treatment with isoniazid, rifampin, pyrazinamide, and ethambutol.

**FIGURE 2:**
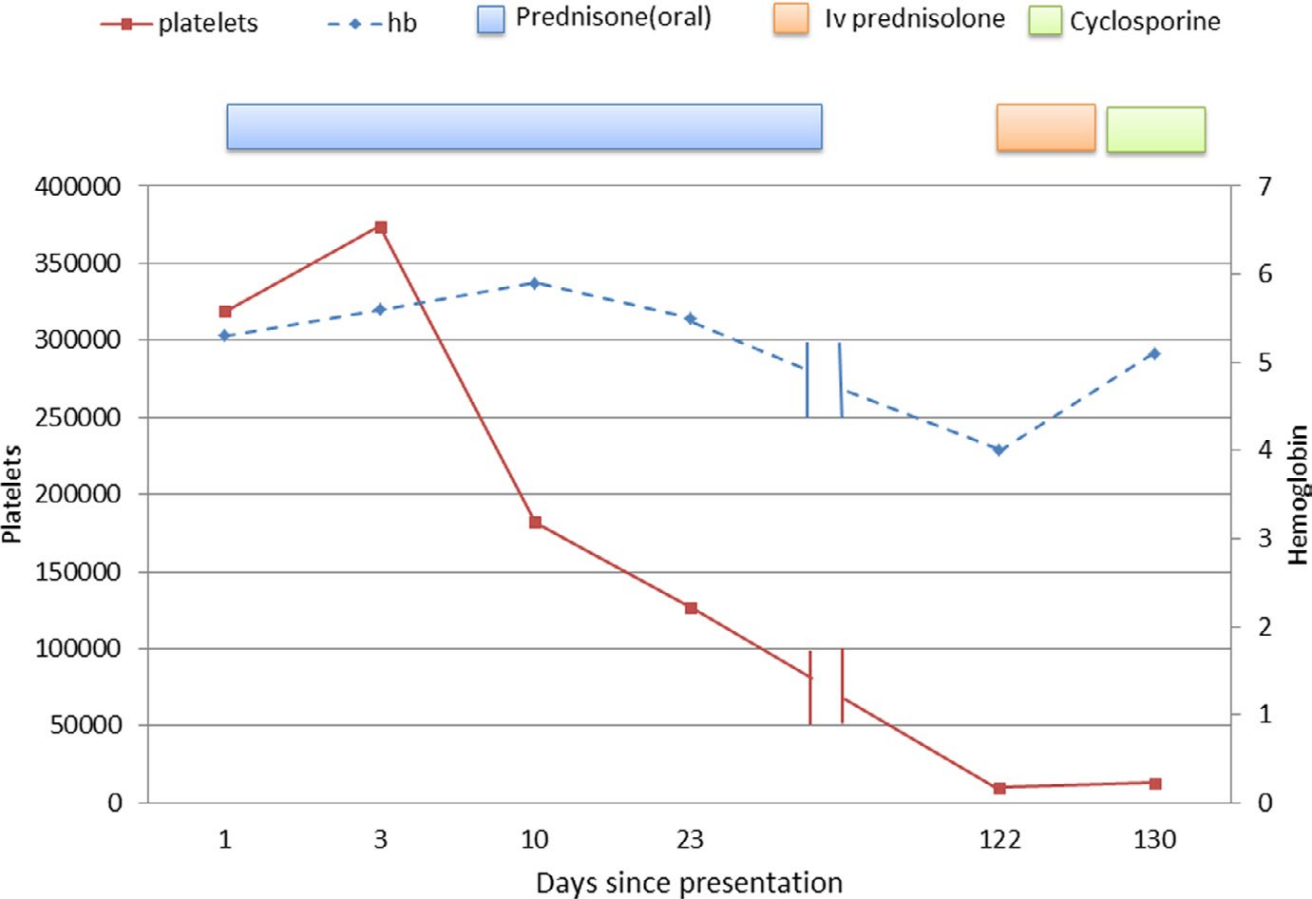
Line diagram showing the hemoglobin and platelets levels since the patient presentation

Follow‐up laboratories showed Hb stable at 5‐7 g/dL. Platelet count decreased to 127 000/mm^3^ at the time of discharge (Figure 2). Following discharge on antitubercular medications and prednisone, he was lost to follow up.

The patient presented to our clinic after 3 months following an exacerbation of his symptoms after the inadvertent stopping of his prednisone. He presented with increased fatigue, generalized weakness, and jaundice. He had no preceding upper respiratory tract symptoms. Blood investigation this time showed Hb: 4 g/dL, WBC: 6500/mm^3^, and platelets: 10 000/mm^3^. LFT showed total bilirubin: 48.05 μmol/L and direct bilirubin: 13.51 μmol/L. LDH was elevated to 851 U/L.

The development of thrombocytopenia in a patient with pre‐existing autoimmune hemolytic anemia led us to consider the possibility of Evans syndrome as the cause of bicytopenia. He was started on intravenous methylprednisolone 1 g once daily for 3 days, followed by oral prednisone 60 mg daily. However, there was no improvement in his Hb or platelet count. We considered other immunosuppressive therapies like intravenous immunoglobulin, rituximab, and cyclosporine as possible treatment options, but we faced two challenges with their use: fear of reactivation of TB and the prohibitive cost. Eventually, we decided to start him on cyclosporine, given its affordable cost despite the risk of reactivation of TB. He was discharged on oral cyclosporine 10 mg daily for a month. Unfortunately, he was again lost to follow up.

## 3 DISCUSSION

Evans syndrome is an autoimmune disorder characterized by the combination of AIHA and immune thrombocytopenia.^1^ Although the worldwide incidence of ES has not been reported in the literature, a French national observational study done in 265 patients with AIHA showed that 37% of the patients had ES, while Pui et al reported that 73% in a cohort of 15 children with AIHA had ES.^4^, ^5^

The pathophysiology underlying ES is not clearly defined but is most likely related to a generalized dysregulation of the immune system, involving both the cellular and humoral immunity.^6^, ^7^ Downregulation of the T‐cell control over the autoreactive B‐cell clones results in a deranged Th1/Th2 ratio with subsequent increased production of IL‐10 and INF‐γ, and decreased generation of TGF‐β. The increased secretion of INF‐ γ (a Th1 cytokine) stimulates the autoimmune B‐cell clones to produce autoantibodies against red cell‐specific and platelet‐specific antigens.^8^ ES is also seen in the background of autoimmune lymphoproliferative syndrome (ALPS), common variable immunodeficiency (CVID), 22q11.2 deletion syndrome, and IgA deficiency, indicating immunodeficiency as a possible predisposing factor for this autoimmune phenomenon.^9^, ^10^, ^11^

Few case reports have described the co‐occurrence of TB and ES.^12^, ^13^, ^14^ Sharma et al reported a case of ES presumed to be secondary to TB. The authors hypothesize that the occurrence of ES in a TB patient may be due to production of antibodies against the blood cells by lymphocytes in response to the tubercular pathogen. Molecular mimicry involving unknown antigens of tubercular bacilli, and platelet surface antigens could be responsible for thrombocytopenia seen in ES patients with TB.^13^ Kim et al reported a case of tuberculosis cutis orificialis in a patient with pre‐existing ES. They discussed the possibilities of impaired cellular immunity and the long‐term use of immunosuppressive medications in ES as predisposing factors for TB.^14^ Hence, ES could predispose to TB and vice versa. In our case, the patient presented with symptoms of TB and ES concurrently. It is possible that a common, yet to be determined, pathophysiological denominator could be responsible for the coexistence of these two seemingly disparate conditions.

Frequent relapses characteristic of ES make its treatment an uphill task. There have been no randomized controlled trials comparing the effectiveness of different modalities of treatments for ES. Corticosteroids have been the mainstay of treatment based on studies from small cohorts, although frequent relapses have been reported. Pui et al, in his study cohort, reported remission with corticosteroid therapy in all six children who required treatment. However, relapse was reported during viral infections or on tapering of the corticosteroid dose.^5^ Those who fail to respond or require a high dose of corticosteroids have reportedly been treated with IVIG.^15^ Other treatment options that have been used for refractory cases include immunosuppressants, blood transfusion, splenectomy, and hematopoietic stem cell transplant.^2^

Further research is needed to unravel the pathophysiology behind the concurrence of ES and TB, so that common pathophysiological culprit, if any, could be targeted. As mentioned above, there are no well‐validated guidelines for the treatment of ES and, by extrapolation, for concurrent ES and TB. The coincidence of TB with ES adds a layer of complexity as the treatment of ES exacerbates TB. Furthermore, the manner in which antitubercular medications, with their nuclear targets, affect the course of ES remains to be studied. Thus, there is a significant knowledge gap in our understanding of this condition. The impetus for research, the locus of which is primarily situated in developed countries, could be dampened as TB is mostly a third world problem. Furthermore, the cost‐effectiveness of therapy should also be an important consideration while devising therapeutic interventions for concurrent TB and ES.

## 4 CONCLUSION

We report a rare case of co‐occurrence of TB and ES. The exact pathophysiology behind the concurrence remains to be elucidated. We believe that ES and TB could be predisposing factors for one another, or there may be a common pathophysiological denominator for the co‐occurrence. Further research is needed for a better understanding of pathophysiology and treatment.

## CONFLICT OF INTEREST

None declared.

## AUTHOR CONTRIBUTION

SG: involved in conception and design, collected the data, drafted the manuscript, and finally approved the version to be published. UJ: involved in conception and design, drafted the manuscript, revised the manuscript, and finally approved the version to be published. SK and ZK: collected the data, drafted the manuscript, and finally approved the version to be published. AS: involved in the revision of manuscript, supervised the manuscript, and finally approved the version to be published.

## ETHICAL APPROVAL

Ethical approval was not required.

## Data Availability

The authors confirm that the data supporting the findings of this study are available within the article.
